# Lactate Dehydrogenase Inhibition Reverts the Fatty Acid‐Induced Neurotoxic Phenotype of Astrocytes

**DOI:** 10.1002/glia.70136

**Published:** 2026-01-06

**Authors:** Daniel Esteve, Mariana Bresque, Daniel Okhuevbie, Sandhya Ramachandran, Mariana Pehar, Marcelo R. Vargas

**Affiliations:** ^1^ Department of Neurology University of Wisconsin‐Madison Madison Wisconsin USA; ^2^ Division of Geriatrics and Gerontology, Department of Medicine University of Wisconsin‐Madison Madison Wisconsin USA; ^3^ Geriatric Research Education Clinical Center Veterans Affairs Medical Center Madison Wisconsin USA

**Keywords:** astrocytes, inflammation, LDH, lipid droplets, NF‐κB

## Abstract

Astrocytes are central to lipid metabolism in the central nervous system. Due to their morphological and functional characteristics, astrocytes can uptake fatty acids (FAs) from the bloodstream and extracellular space and store them in lipid droplets (LD). LD are dynamic organelles, whose accumulation in astrocytes has been shown to occur upon exposure to various stress stimuli. Different hypotheses proposed to explain motor neuron degeneration in amyotrophic lateral sclerosis (ALS) implicate mitochondrial dysfunction and oxidative stress. Mitochondrial dysfunction in astrocytes is associated with elevation of cytoplasmic lipids and lipid‐binding proteins. We observed increased LD in the spinal cord of symptomatic ALS mice, as well as in human transdifferentiated astrocytes obtained from ALS patients. Using a co‐culture model, we examined the effect of FA overload and its impact on astrocyte–motor neuron interaction. LD accumulation was tightly coupled with an NF‐κB‐driven proinflammatory response in nontransgenic astrocytes, correlating with motor neuron toxicity. These results provide additional evidence to the notion that altered energy balance may contribute to neuronal death in ALS. Furthermore, pharmacological inhibition of lactate dehydrogenase (LDH) reversed LD accumulation in mouse and human astrocytes expressing ALS‐linked mutations. Genetic ablation of LDHA similarly reduced LD accumulation in response to FA treatment. Collectively, our data underscore the role of lipid metabolism in astrocyte–neuron interactions in ALS models and suggest that LD accumulation, rather than serving solely as a protective mechanism, reflects a metabolic stress state linked to a detrimental phenotypic transformation in astrocytes.

## Graphical abstract

- ALS models display increased LD accumulation.
- LD accumulation is linked to a neurotoxic phenotype in astrocytes.
- Lactate dehydrogenase inhibition reverts LD accumulation in ALS astrocytes.

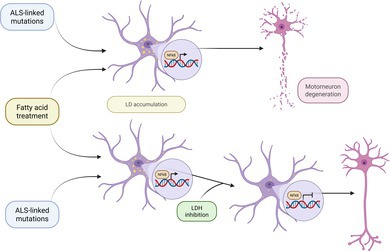

## 1 Introduction

Astrocytes play a major role in lipid metabolism in the central nervous system (CNS). Due to their morphological and functional characteristics, astrocytes can uptake fatty acids (FAs) from the bloodstream and extracellular space and store them in lipid droplets (LD). LD are dynamic organelles that store FAs as triacylglycerols (TAG) and sterol esters. The biogenesis of cytoplasmic LD is believed to occur in the endoplasmic reticulum (ER), starting with the esterification of an activated FA through the activity of acetyl‐CoA acetyltransferases (ACAT1 and ACAT2) and diacylglycerol transferases (DGAT1 and DGAT1) (Zadoorian et al. 2023). The result is a core of neutral lipids surrounded by a phospholipid monolayer with some components from the ER bilayer. In this monolayer, LD‐associated surface proteins control the dynamics of LD. Among them, proteins of the perilipin family, with subtypes that vary according to the cell type, are essential in the formation and degradation of LD (Itabe et al. 2017).

While changes in the number of LD have been best characterized in adipocytes and hepatocytes as a side effect of metabolic disease (Farese Jr. and Walther 2009), LD have important functions beyond energy homeostasis. Recent studies have highlighted the importance of LD in neuronal and glial cells, where they can react dynamically to various stimuli. For example, astrocytes can buffer an excess of FAs under oxidative stress conditions, preventing lipotoxicity and subsequent negative consequences for neighboring neurons (Chitraju et al. 2017; Goodman et al. 2024; Liu et al. 2015, 2017; Olzmann and Carvalho 2019). In addition, mitochondrial dysfunction in astrocytes also triggers LD accumulation, followed by neurodegeneration and neuroinflammation (Mi et al. 2023; Young et al. 1996). It appears that LD act as a hub that coordinates cellular metabolism with key cellular functions critical for CNS health. Thus, it is not surprising that changes in LD biology have been linked to neurodegenerative diseases like Alzheimer's disease and amyotrophic lateral sclerosis (ALS) (Ralhan et al. 2021).

ALS is characterized by progressive neurodegeneration in the motor cortex, brainstem, and spinal cord. The molecular mechanism responsible for motor neuron degeneration in ALS remains uncertain. However, evidence obtained from numerous in vitro and in vivo models of ALS has pointed toward a role of astrocytes shaping motor neuron fate (Boillee et al. 2006; Clement et al. 2003; Di Giorgio et al. 2007; Ditsworth et al. 2017; Foust et al. 2013; Haidet‐Phillips et al. 2011; Kia et al. 2018; Meyer et al. 2014; Nagai et al. 2007; Vargas et al. 2006; Wang et al. 2011; Yamanaka et al. 2008). Many of the different hypotheses proposed to explain motor neuron degeneration in ALS implicate mitochondrial dysfunction and oxidative stress, either as a primary cause or as a secondary component of the pathogenic process (Cozzolino et al. 2013; Pehar et al. 2017; Shi et al. 2010). Mitochondrial dysfunction in astrocytes is associated with an increase in cytoplasmic lipids and lipid‐binding proteins (Bailey et al. 2015; Liu et al. 2015, 2017) and could be responsible for the changes in the lipidome profile observed in the spinal cord of ALS mouse models (Chaves‐Filho et al. 2019). In addition, the existence of changes in LD dynamics has been used to suggest dysregulated metabolism in astrocytes expressing ALS‐linked mutant proteins (Jimenez‐Riani et al. 2017; Miquel et al. 2024; Velebit et al. 2020).

Based on these observations, a link between dynamic changes in LD and the development of ALS pathology has been suggested (Pennetta and Welte 2018). However, to the best of our knowledge, in vivo evidence of altered LD biology in ALS models has not been presented so far. In this study, we investigate the presence of LD in different ALS models, both in vivo and in vitro. In addition, we analyze the effect of increasing the number of LD in the biology of astrocytes. We observed that in spinal cord astrocytes, LD accumulation following FA treatment induces inflammation and confers upon astrocytes a neurotoxic phenotype. Furthermore, we show that metabolic reprogramming of astrocytes through the inhibition of lactate dehydrogenase (LDH) reverts this neurotoxic phenotype. Taken together, our data reflect potential implications of lipid metabolism in astrocyte–neuron interaction in ALS pathology.

## 2 Materials and Methods

### 2.1 Animals

All animal procedures were approved by the UW‐Madison Animal Care and Use Committee and performed according to the National Institutes of Health Guidelines for the Care and Use of Laboratory Animals. Mice were housed in a temperature and humidity‐controlled room with a 12‐h light and 12‐h dark cycle with ad libitum access to water and standard diet. B6.Cg‐Tg(SOD1G93A)1Gur/J, B6.Cg‐Tg(SOD1)2Gur/J (Gurney et al. 1994) and B6(Cg)‐Ldha^tm1c(EUCOMM)Wtsi^/DatsJ (Wang et al. 2014) were obtained from the Jackson Laboratory and maintained as hemizygous animals in a C57BL/6J background. B6.Cg‐Tg(SOD1)2Gur/J mice express human wild‐type SOD1 at similar levels to the mutant human SOD1 in B6.Cg‐Tg(SOD1G93A)1Gur/J mice. Human SOD1^H46R/H48Q^ mice were provided by Dr. David Borchelt (Wang et al. 2002) and have been backcrossed into C57BL/6J pure background for more than 10 generations.

### 2.2 Primary and iPSCs Cultures

Neonatal primary astrocyte cultures were prepared from the spinal cord of 1‐day‐old mice as previously described (Vargas et al. 2006). Primary spinal cord astrocyte cultures from symptomatic hSOD1^G93A^ and age‐matched nontransgenic mice were prepared from individual spinal cord as previously described (Killoy et al. 2020). Astrocytes isolated from adult mice were plated in Matrigel (Corning, CLS354277)‐coated plates. Experiments were performed when astrocytes reached 100% confluency after approximately 4 weeks. Motor neuron cultures were prepared from 12.5‐embryonic‐day mouse spinal cords as previously described (Vargas et al. 2008). For co‐culture experiments with neonatal spinal cord astrocytes, motor neurons were plated on mouse astrocyte monolayers at 550 cells/cm^2^ density and maintained in a supplemented L15 medium (Vargas et al. 2006). For co‐culture experiments with spinal cord astrocytes from symptomatic mice, motor neurons were plated on astrocyte monolayers at 800 cells/cm^2^ density and maintained in supplemented neurobasal medium (Killoy et al. 2020). Motor neurons were identified by immunostaining with an anti‐βIII‐Tubulin antibody (Millipore, 05‐559), and the survival of motor neurons was determined by counting βIII‐Tubulin‐positive cell bodies displaying intact neurites longer than four cell bodies in diameter. Counts were performed over an area of 0.90 cm^2^ in 24‐well plates (Killoy et al. 2020).

iPSC lines from ALS‐patients and isocorrected controls were obtained from the Cedars Sinai Biomanufacturing Center (CS29iALS‐C9n1 and CS29iALS‐C9n1.ISOT2RB4) or the WiCell institute (WC034i‐SOD1‐D90A and WC035i‐SOD1‐D90D). iPSCs were differentiated into induced NPCs using an embryoid body formation protocol in the presence of SMAD signaling inhibitors (STEMdiff SMADi Media, Stemcell). Induction was confirmed by an increase in MAP2, PAX6, and NESTIN gene expression and a concurrent decrease in SOX2, OCT3, and NANOG expression. Induced NPCs were cultured for 3 weeks in astrocyte differentiation media (STEMdif Astrocyte Differentiation Media, Stemcell), followed by 3 weeks in astrocyte maturation media (STEMdif Astrocyte Maturation Media, Stemcell). Astrocyte differentiation was confirmed by assessing GFAP, S100B, and ALDH1L1 gene expression. Following differentiation, induced astrocytes were cultured in DMEM‐F12 supplemented with 10% FBS and 0.3% N2 supplement.

### 2.3 Cell Treatments

LD formation was induced using oleic acid (OA)–bovine serum albumin (BSA) (Sigma, #O3008) and linoleic acid (LA)–BSA (Sigma, #L9530) for 24 h at 50 and 100 μM concentrations. BSA was used as vehicle control. To evaluate the potential cytotoxic effect of the FA treatments, the CytoTox96 Non‐Radioactive Cytotoxicity Assay (Promega) was used following the manufacturer's instructions. No toxicity was observed in spinal cord astrocyte cultures after treatment with the indicated concentrations of OA and LA for 24 h. LDHA inhibition was performed 24 h after FA treatment with the selective inhibitor GSK2837808A (Millipore) at 2.5 μM for 24 h. Carnitine palmitoyltransferase 1 (CPT1) inhibition was achieved using Etomoxir (Sigma, 5094550001) at 10 μM, 24 h after FA treatment and 4 h before the LDHA inhibition. Transduction with a Cre recombinase expressing adenovirus (Ad‐CMV‐Cre, Vector Biolabs #1045) or a control adenovirus containing an empty CMV promoter (Ad‐CMV‐Null, Vector Biolabs #10300) was performed at 5 M. O. I. 72 h before FA treatments.

### 2.4 Immunofluorescence

Astrocyte cultures were fixed with 4% PFA and 0.1% glutaraldehyde in PBS for 20 min, followed by permeabilization with 0.1% Triton X‐100 in PBS. Nonspecific binding was blocked with 10% goat serum, 2% BSA, and 0.1% TritonX‐100 in PBS for 1 h at room temperature. Fixed astrocyte cultures were incubated overnight at 4°C with the primary antibodies: NF‐κB p65 (D14E12) XP Rabbit mAb (Cell Signaling, #8242), and/or GFAP Antibody (GA5) (Novus, #NBP2‐29415). Secondary antibodies were as follows: Alexa Fluor 488‐conjugated goat anti‐mouse (Invitrogen, #A11029), Alexa Fluor 594‐conjugated goat anti‐mouse (Invitrogen, #A11032), or Alexa Fluor 594‐conjugated goat anti‐rabbit (Invitrogen, #A11037). LD were stained with LipidGreen2 (Millipore, #5.06029.0001) and nuclei were counterstained with DAPI (4′,6‐Diamidino‐2‐phenylindole dihydrochloride). LD in astrocyte cultures were quantified from at least three images per well in each independent experiment, using the software ImageJ and normalized by the number of nuclei. Analysis of LD formation in vivo was performed in frozen sections of lumbar spinal cords from nontransgenic and symptomatic ALS mice. Briefly, after perfusion with PBS, spinal cord tissue was fixed in 10% formalin, cryoprotected with 30% sucrose in PBS and cryosectioned at 20 μm. The cryosections were permeabilized with 0.1% Triton X‐100 in PBS and blocked with 10% donkey serum, 2% BSA, and 0.1% Triton X‐100 in PBS. Primary antibodies used were: GFAP Antibody (GA5) (Novus, #NBP2‐29415) and Perilipin‐2/ADFP antibody (Novus, #NB110‐40877). Secondary antibodies were as follows: Alexa Fluor 594 AffiniPure Donkey Anti‐Rabbit IgG (H + L) and Alexa Fluor 647 AffiniPure Donkey Anti‐Mouse IgG (H + L) (Jackson ImmunoResearch). LD were stained with LipidGreen2 and nuclei were counterstained with DAPI. Images were acquired using a Nikon AXR confocal microscope with identical acquisition settings across all sections. *Z*‐stacks were captured with a step size of 0.5 μm. LD quantification was performed using ImageJ software. First, the 10‐slice *z*‐stacks were converted into a single plane using the *Z*‐sum slices projection function. Background noise was removed using a rolling ball radius of 50 pixels. To identify LD, the Perilipin‐2 and LipidGreen2 channels were merged, and a threshold range of 20–255 was applied. Particles were defined as LD if they had a size greater than 1.5 μm^2^ and a circularity between 0.5 and 1.0. Quantification was performed within a 50,000 μm^2^ region of interest (ROI) located in the ventral horn of the spinal cord. Data were derived from at least four tissue sections per animal.

### 2.5 Western Blot Analysis

Western blot membranes were incubated overnight with one of the following antibodies: Perilipin‐2/ADFP antibody (Novus, #NB110‐40877), FABP7 (Sigma, ZRB13190), LDHA (Sigma, SAB5700695), and ACTIN (Sigma, #A5441). Image acquisition was performed in an ImageQuant 500 western blot imaging system (Cytiva). Quantifications were performed using the Image Studio Software (Li‐Cor).

### 2.6 ELISA Assays

CXCL10 and TNFα levels in astrocyte‐conditioned media were determined using a mouse CXCL10/IP‐10/CRG‐2 ELISA kit and mouse TNF‐alpha DuoSet ELISA kit, respectively (R&D Systems).

### 2.7 NF‐κB Reporter Assay

Adenovirus expressing a firefly luciferase gene under the control of a synthetic promoter that contains direct repeats of the NF‐κB binding site (Ad‐NFkb‐Luc) or a *Renilla* luciferase under a constitutive promoter (Ad‐pRL‐Luc) were obtained from Vector Biolabs. Transductions of astrocytes and luciferase assays were performed as previously described (Killoy et al. 2020).

### 2.8 Statistical Analysis

Groups of three to four animals were used for histological analysis. Multiple tissue sections per animal were analyzed. The data points depicted in the graphs correspond to the data obtained in each section. For cell culture, unless otherwise indicated, each experiment was repeated in at least three independent primary culture preparations, and values from each experimental replicate are presented for data reporting. For image analysis, at least three pictures from each well in an experiment were used for quantification. All data are reported as mean ± SD. Comparisons between two groups were performed by unpaired *t*‐tests. Multiple group comparisons were performed by one‐way analysis of variance (ANOVA) with Tukey's posttest. When comparing the effect of genotype and treatments, two‐way ANOVA was used, followed by Tukey's posttest. Differences were declared statistically significant if *p* ≤ 0.05. All statistical computations were performed using Prism 10 (GraphPad Software).

## 3 Results

**FIGURE 1:**
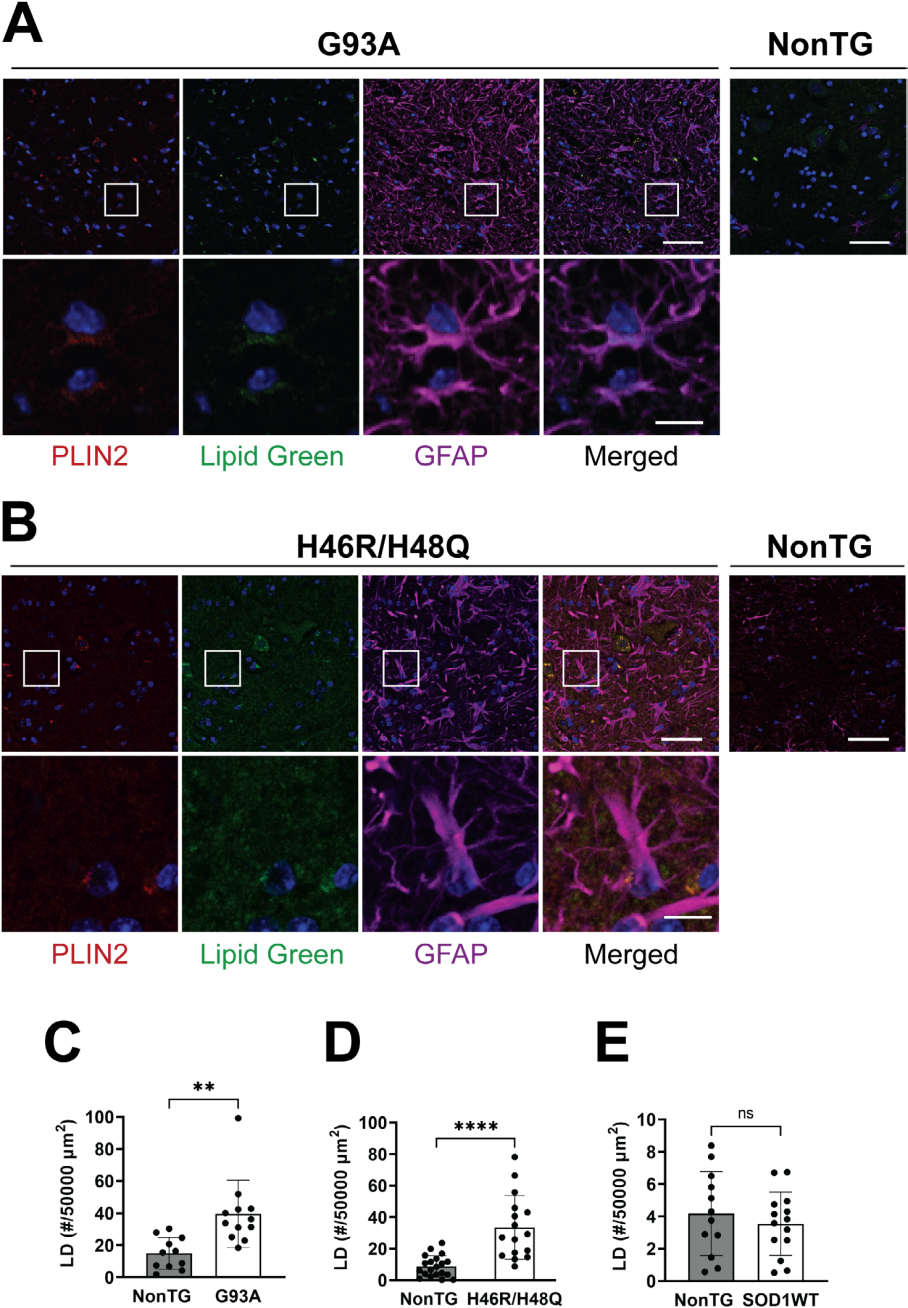
Increased lipid droplet content in the ventral horn of the spinal cord from SOD1‐linked ALS mouse models. Perilipin 2 (PLIN2, red), LipidGreen2 (green), and GFAP (magenta) staining in lumbar spinal cord sections of age‐matched nontransgenic (NonTG), symptomatic hSOD1^G93A^ (G93A) (A) and symptomatic hSOD1^H46R/H48Q^ (H46R/H48Q) (B) mice. Only the merged image is shown for NonTG mice. Nuclei were counterstained with DAPI (blue). The inset highlights GFAP‐positive cells with LD, inset amplification is shown at the bottom of each panel. Images were acquired at an exposure setting that prevents saturation of GFAP staining in ALS mice. The intensity of GFAP staining in NonTG mice is lower compared to the intensity of staining in ALS mice, and thus not clearly detected at the settings used. Scale bar: 50 and 10 μm for the inset amplification. Quantification of the number of LD present in 50,000 μm^2^ area of the ventral horn in the spinal cord from age‐matched NonTG and symptomatic G93A (C), symptomatic H46R/H48Q (D), and wild‐type hSOD1 (SOD1WT) (E) mice (*n* = 3, mean ± SD). ****p < 0.0001, **p < 0.01.

**FIGURE 2:**
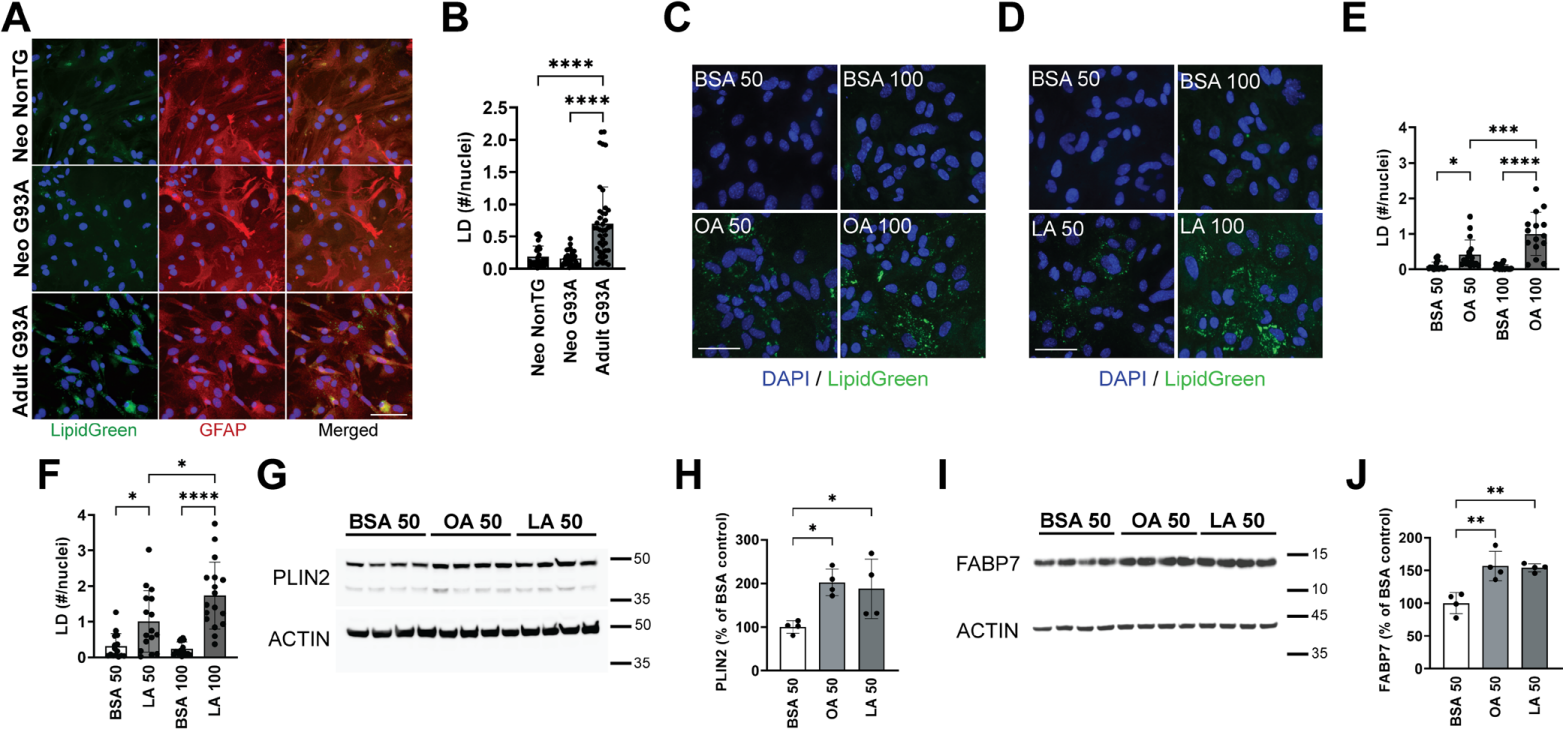
Accumulation of LD in spinal cord astrocyte cultures from symptomatic hSOD1^G93A^ mice and nontransgenic mice treated with fatty acids. (A) LD (LipidGreen2, green) and GFAP (red) staining in spinal cord astrocyte cultures from neonatal (Neo) NonTG and hSOD1^G93A^ (G93A) mice and from symptomatic (adult) G93A mice. Nuclei were counterstained with DAPI (blue). Scale bar: 50 μm. (B) Quantification of LD normalized by the number of nuclei in the above cultures (*n* = 3, mean ± SD). (C and D) Representative fluorescence images showing LD staining (LipidGreen2, green) in neonatal NonTG spinal cord astrocytes cultures treated for 24 h with oleic acid (OA) (C) or linoleic acid (LA) (D), at a concentration of 50 μM or 100 μM. Control cultures were treated with BSA (used as a carrier for FAs), at the corresponding concentration. Nuclei were counterstained with DAPI (blue). Scale bar: 50 μm. (E and F) Quantification of LD in spinal cord astrocyte cultures treated with OA and LA after normalization by the number of nuclei (*n* = 3–4, mean ± SD). (G and H) Western blot analysis of Perilipin 2 (PLIN2) expression in neonatal spinal cord astrocyte cultures from NonTG mice treated with vehicle (BSA), OA (50 μM), or LA (50 μM). Quantification of PLIN2 levels is shown in (H). PLIN2 expression was quantified, normalized by Actin levels, and expressed as a percentage of cultures treated with vehicle (BSA) (*n* = 4, mean ± SD). (I and J) Western blot analysis of fatty acid binding protein 7 (FABP7) expression in neonatal spinal cord astrocyte cultures from NonTG mice treated as indicated above. Quantification of FABP7 levels is shown in (J). FABP7 expression was quantified, normalized by actin levels, and expressed as a percentage of cultures treated with vehicle (BSA) (*n* = 4, mean ± SD) ****p < 0.0001, ***p < 0.001, *p < 0.05.

To investigate altered LD dynamics in ALS, we first analyzed lumbar spinal cord sections of two different SOD1‐linked ALS mouse models after immunostaining against the LD marker Perilipin 2 (PLIN2) and co‐staining with LipidGreen2. We observed a significant increase in the number of LD in the ventral horn of the spinal cord of both hSOD1^G93A^ and hSOD1^H46R/H48Q^ early symptomatic mice, when compared with their age‐matched nontransgenic controls (Figure 1). In the spinal cord of transgenic mice that overexpress human wild‐type hSOD1, which do not develop overt motor neuron degeneration, the number of LD remains unchanged (Figures 1E and S1). The accumulation of LD in the ventral horn of the spinal cord of ALS mice occurs in GFAP‐positive astrocytes as well as in GFAP‐negative cells. While these GFAP‐negative, LD‐containing cells likely represent microglia and neurons, our study focuses specifically on astrocytes. Since LD dynamics in astrocytes have been suggested to affect neighboring neurons (Chitraju et al. 2017; Goodman et al. 2024; Liu et al. 2015, 2017; Olzmann and Carvalho 2019), we investigated the presence of LD in primary astrocytes isolated from neonatal and symptomatic hSOD1^G93A^ mice. While primary astrocytes isolated from neonatal ALS mice did not show an increased number of LD, astrocyte cultures obtained from the spinal cord of symptomatic hSOD1^G93A^ displayed a greater number of LD when compared to nontransgenic astrocytes (Figure 2A,B).

Cultured astrocytes can uptake excessive exogenous FAs and store them in LD (Nakajima et al. 2019; Smolic et al. 2021). To study the effect of LD accumulation in spinal cord astrocytes, we treated confluent primary spinal cord astrocyte cultures from nontransgenic mice with oleic acid (OA) and linoleic acid (LA) for 24 h. We selected OA and LA based on their distinct biochemical properties and physiological prevalence in the CNS. Changes in both these FAs have been observed during neuronal damage, and astrocytes may be exposed to high local concentrations of these FAs during these events. OA, a monounsaturated FA, is the primary FA constituent of myelin sheaths. Following CNS injury, myelin degradation leads to a surge in local extracellular OA concentrations (Martinez and Mougan 1998; Nessel and Michael‐Titus 2021). In addition, OA is a proven inducer of LD formation in astrocytes. In contrast, LA, an omega‐6 polyunsaturated FA (PUFA), serves as the metabolic precursor to arachidonic acid. This pathway is critical for the synthesis of proinflammatory eicosanoids, which are elevated in the acute phase of CNS injury, and it is likely that LA is rapidly stored into LD to prevent lipotoxicity and peroxidation (Dhillon et al. 1997; Hennebelle et al. 2017; Pilitsis et al. 2003). As demonstrated in Figure 2, both treatments induced the accumulation of LD (Figure 2C–F), which was paralleled by a significant increase in both PLIN2 and fatty acid binding protein 7 (FABP7) expression (Figure 2G–J). PLIN2 protein expression is markedly upregulated by FAs (Gao et al. 2000), while FABP7 expression has been shown to protect astrocytes from noxious stimuli through the promotion of LD formation (Islam et al. 2019). Thus, the upregulation of both proteins provides additional evidence of an increase in LD content in astrocytes following excess FA treatment.

**FIGURE 3:**
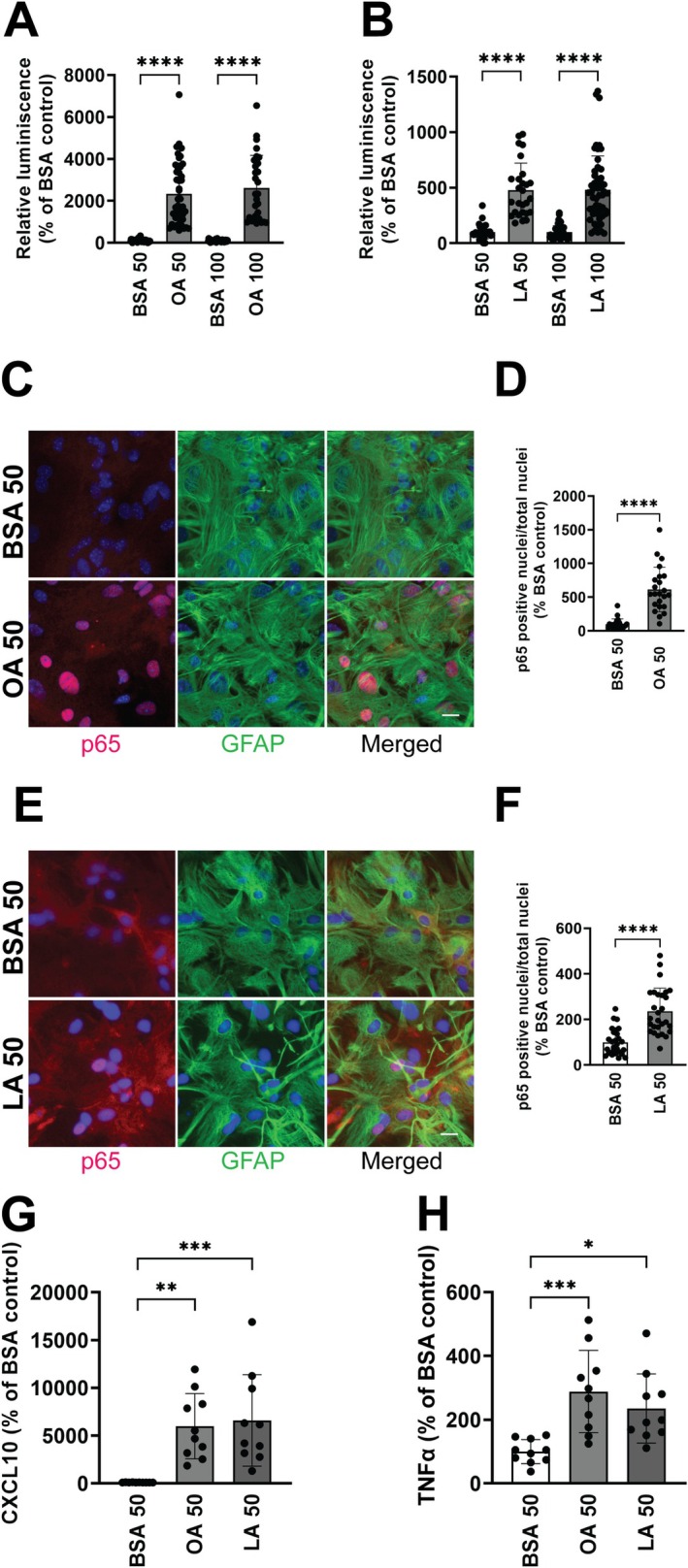
Fatty acid treatment induces a proinflammatory phenotype in astrocytes. Relative luminescence produced by firefly luciferase expressed under the control of an NF‐κB‐driven promoter after 24 h of treatment with (A) oleic acid (OA, 50 and 100 μM) and (B) linoleic acid (LA, 50 and 100 μM), in nontransgenic neonatal spinal cord astrocytes cultures. Control cultures were treated with BSA (used as a carrier for FAs). Relative firefly luciferase luminescence was corrected by the amount of *Renilla* luciferase activity controlled by a constitutive promoter and expressed as a percentage of vehicle (BSA)‐treated cultures (*n* = 3, mean ± SD). (C–F) Representative images depicting immunostaining against NF‐κB‐p65 (red) and GFAP (green) in neonatal spinal cord astrocytes 4 h after treatment with (C) OA (50 μM) or (E) LA (50 μM). Nuclei were counterstained with DAPI (blue). Scale bar: 25 μm. Quantification of NF‐κB‐p65‐positive nuclei in neonatal spinal cord astrocytes 4 h after treatment with (D) OA (50 μM) or (F) LA (50 μM). Data are expressed as NF‐κB‐p65 positive nuclei over the total number of nuclei analyzed, percentage of vehicle (BSA)‐treated cultures (*n* = 5, mean ± SD). (G and H) ELISA quantification of CXCL10 (G) and TNFα (H) levels in conditioned media from astrocytes treated with OA (50 μM) and LA (50 μM). Data are expressed as percentage of vehicle (BSA)‐treated control cultures (*n* = 2, 5 treatment replicates per experiment, mean ± SD). ****p < 0.0001, ***p < 0.001, **p < 0.01, *p < 0.05.

**FIGURE 4:**
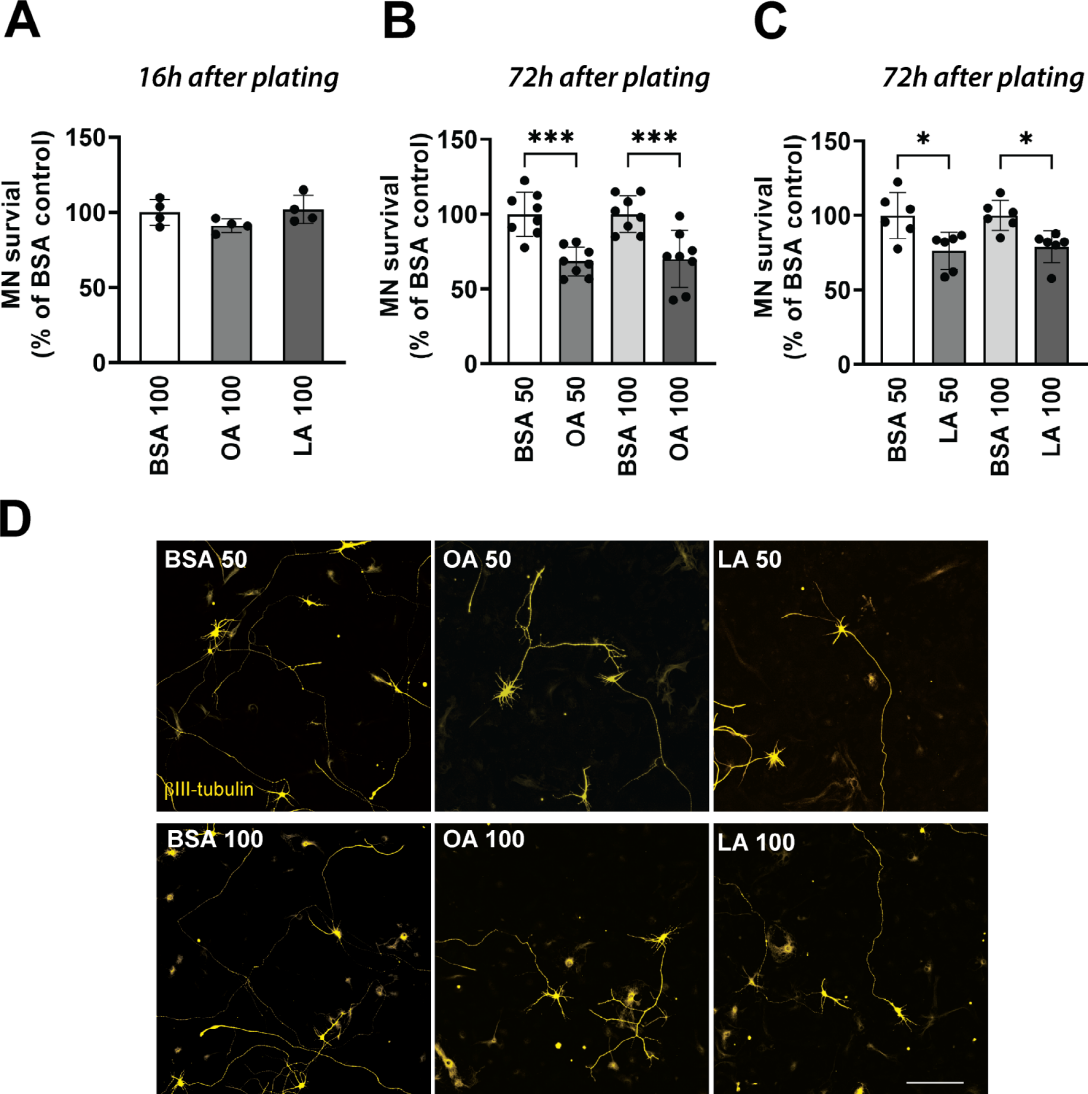
Fatty acid treatment induces a neurotoxic phenotype in nontransgenic astrocytes. (A) Spinal cord nontransgenic astrocytes were treated with 100 μM oleic acid (OA) or linoleic acid (LA) and 24 h later embryonic motor neurons were plated on top. Motor neuron (MN) survival was determined 16 h later (*n* = 2, mean ± SD). The same co‐culture setup as in (A) but motor neuron survival was determined 72 h later and the astrocytes were treated with (B) OA (50 and 100 μM) or (C) LA (50 and 100 μM) (*n* = 3, mean ± SD). *p < 0.05, ***p < 0.001. (D) Representative images of βIII‐Tubulin immunostaining in co‐cultures treated as indicated above. Scale bar: 200 μm.

Multiple non‐cytokine triggers can lead to NF‐κB activation in astrocytes, including metabolic changes affecting FA metabolism (Douglass et al. 2017; Mi et al. 2023; Zhang et al. 2008). While astrocytes may be able to dynamically regulate the number of LD to confer neuroprotection against stress‐induced lipotoxicity (Liu et al. 2015, 2017), we tested if this buffering capacity may eventually be overwhelmed and induce detrimental phenotypic changes. Using a NF‐κB reporter assay, we found that treatment with OA and LA significantly increased NF‐κB‐driven transcriptional activity (Figure 3A,B), which correlated with an increase in the amount of NF‐κB‐p65 nuclear translocation, as determined by immunofluorescence (Figure 3C–F). This increase in NF‐κB‐driven transcriptional activity correlates with an increase in the levels of the proinflammatory cytokines, CXCL10 and TNFa in the conditioned media of treated astrocytes (Figure 3G,H). These results suggest that FA‐treatment and changes in LD dynamics in astrocytes may lead to the acquisition of a proinflammatory phenotype that can be detrimental to neighboring motor neurons. To test this hypothesis, we evaluated the survival of motor neurons in co‐culture with astrocytes pretreated with OA or LA. FA‐treatment does not alter the number of motor neurons that attach to the astrocyte monolayer, since a similar number of motor neurons was observed after 16 h in co‐culture (Figure 4A). However, after 3 days, a decrease in motor neuron survival was observed in co‐cultures with OA‐ or LA‐treated astrocytes, when compared to co‐cultures with vehicle‐treated astrocytes (Figure 4B,C).

**FIGURE 5:**
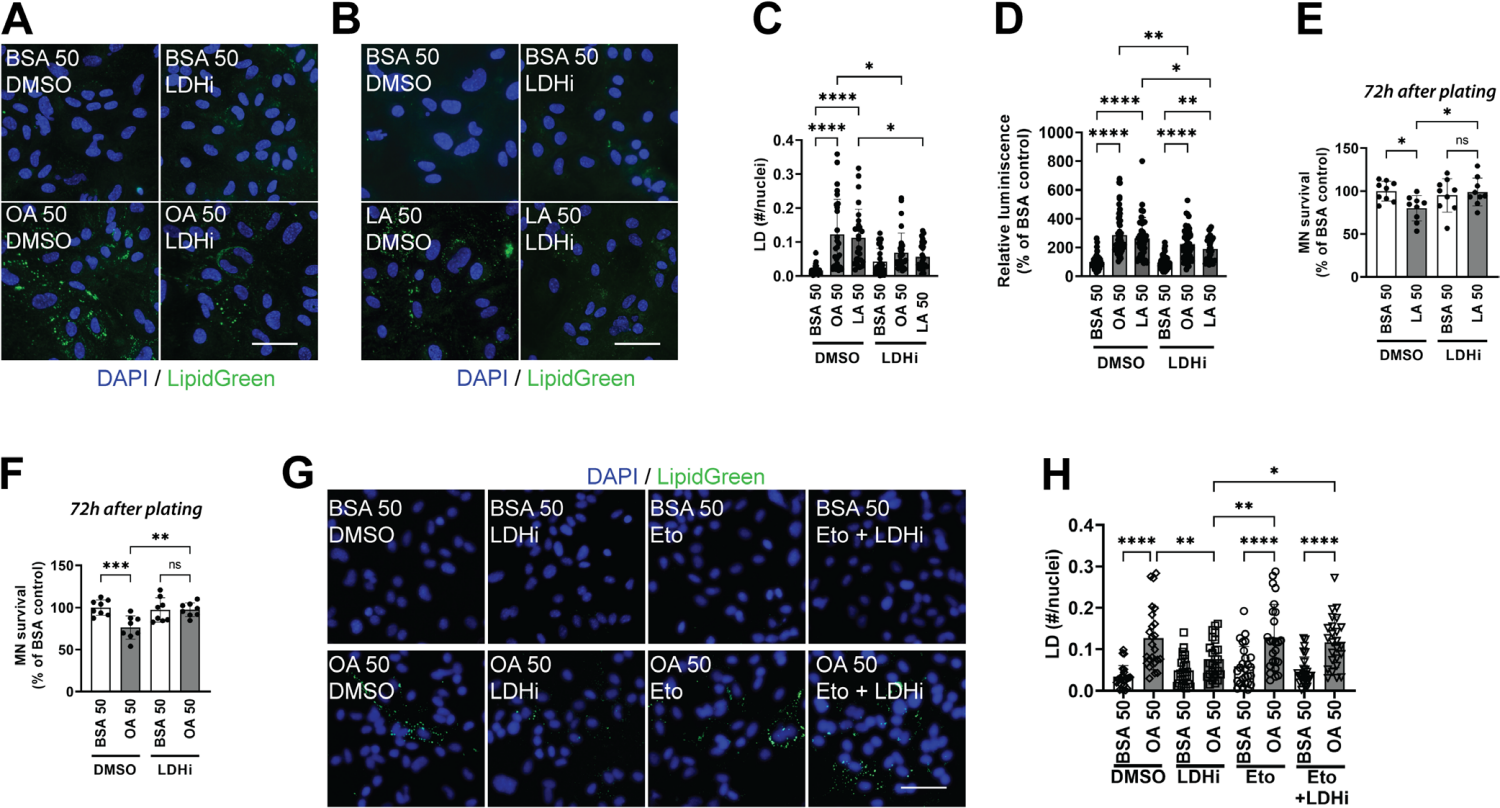
Lactate dehydrogenase inhibition reverts the effect of fatty acid treatment in neonatal spinal cord astrocytes. Neonatal spinal cord astrocytes were treated with vehicle (BSA) or fatty acids for 24 h, followed by treatment with vehicle (DMSO) or GSK2837808A (2.5 μM, LDHi) for 24 h. Representative images of LD staining (LipidGreen2) in spinal cord astrocyte cultures treated with (A) oleic acid (OA, 50 μM) in the presence or absence of LDHi (2.5 μM) and (B) linoleic acid (LA, 50 μM) in the presence or absence of LDHi (2.5 μM). Nuclei were counterstained with DAPI (blue). Scale bar: 50 μm. (C) Quantification of LD in spinal cord astrocytes treated as indicated above. LD numbers were normalized by the number of nuclei analyzed (*n* = 4, mean ± SD). (D) Relative luminescence produced by firefly luciferase expressed under an NF‐κB‐driven promoter in cultures treated as indicated above. Relative firefly luciferase luminescence was corrected by the amount of *Renilla* luciferase activity controlled by a constitutive promoter and expressed as percentage of cultures treated with vehicle (BSA and DMSO) (*n* = 3, mean ± SD). (E and F) Motor neuron survival determined 72 h after being plated on top of astrocytes treated as indicated above (*n* = 4, mean ± SD). (G) Representative images of LD staining (LipidGreen2) in spinal cord astrocyte cultures treated with OA (50 μM) in the presence or absence of LDHi (2.5 μM), Etomoxir (10 μM, Eto), or both combined (Eto + LDHi). Neonatal spinal cord astrocytes were treated with vehicle (BSA) or fatty acids for 24 h, followed by treatment with vehicle (DMSO) or GSK2837808A (2.5 μM, LDHi) and/or Etomoxir (10 μM) for 24 h. Nuclei were counterstained with DAPI (blue). Scale bar: 50 μm. (H) Quantification of LD in spinal cord astrocytes treated as indicated above. LD numbers were normalized by the number of nuclei analyzed (*n* = 3, mean ± SD). ****p < 0.0001, ***p < 0.001, **p < 0.01, *p < 0.05.

Astrocytes in culture predominantly rely on aerobic glycolysis (the conversion of glucose to lactate despite sufficient oxygen availability) for energy production (Dringen and Hamprecht 1993; Supplie et al. 2017; Swanson and Benington 1996). Nonetheless, they retain considerable capacity for mitochondrial oxidative phosphorylation and can readily switch between these metabolic pathways (Arend et al. 2019; Harders et al. 2024). We therefore examined whether inhibition of LDH facilitates metabolic clearance of intracellular lipid stores, a process likely suppressed under glucose‐rich conditions, and mitigates lipid‐induced cellular stress. Moreover, astrocyte activation and the associated enhancement of immune functions are characterized by increased metabolic demand, primarily sustained through aerobic glycolysis and LDH activity (Meyer et al. 2022; Xiong et al. 2022). Thus, pharmacological inhibition of LDH may concurrently promote LD clearance and attenuate the metabolic upregulation required to support a proinflammatory phenotype. Treatment of astrocytes with an LDHA inhibitor 24 h after FA treatment decreases the accumulation of LD (Figure 5A–C). This was paralleled by a 25%–30% decrease in NF‐κB‐driven transcriptional activity when compared to the FA‐treated control group (Figure 5D). Moreover, LDHA inhibition reverted the toxicity of FA‐treated astrocytes toward co‐cultured motor neurons (Figure 5F,G).

Given the metabolic flexibility of astrocytes, we sought to determine if the decrease in LD content following LDH inhibition was dependent on mitochondrial β‐oxidation. The transport of long‐chain FAs into the mitochondria (the rate‐limiting step of β‐oxidation) is controlled by the carnitine palmitoyltransferase 1 (CPT1) family of enzymes (Schlaepfer and Joshi 2020). We therefore tested this pathway using the irreversible CPT1 inhibitor, Etomoxir. Treatment of astrocytes with Etomoxir 4 h prior to LDH inhibition completely abolished the LDH inhibition‐mediated decrease in LD accumulation (Figure 5G,H). These results demonstrate that the reduction in LD content observed upon inhibition of aerobic glycolysis depends on the cell's ability to activate or sustain mitochondrial β‐oxidation.

**FIGURE 6:**
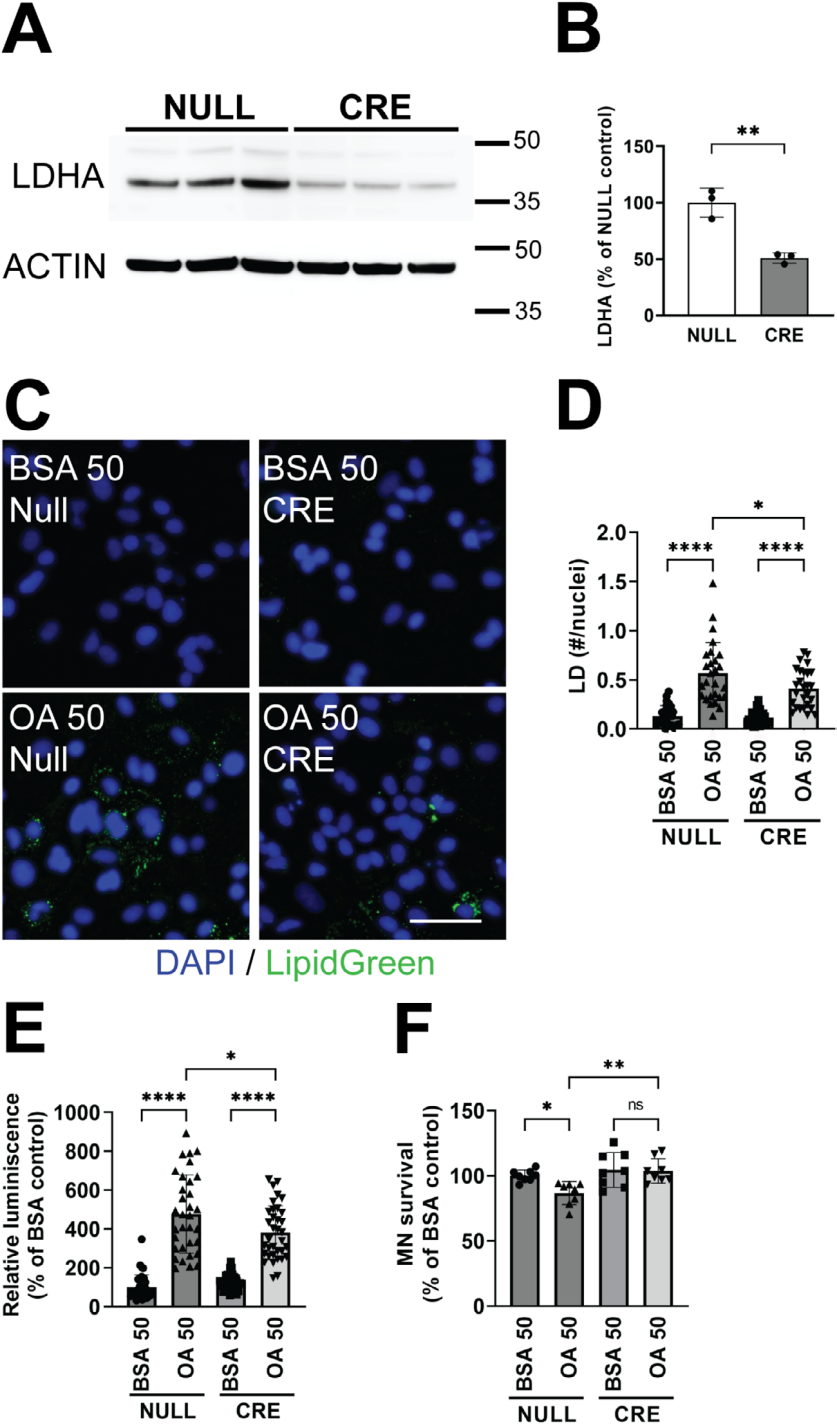
Lactate dehydrogenase gene ablation reverts the effect of fatty acid treatment in neonatal spinal cord astrocytes. (A) Primary confluent spinal cord astrocyte cultures obtained from neonatal Ldha^flox/flox^ mice were transduced with an adenovirus expressing Cre recombinase (CRE) or an empty ORF (NULL). Seventy‐two hours later lactate dehydrogenase A (LDHA) expression was analyzed by Western blot. (B) LDHA expression was quantified, normalized by actin levels, and expressed as a percentage of NULL‐treated cultures (*n* = 3, mean ± SD). (C) Ldha^flox/flox^ spinal cord astrocyte cultures treated as above were subsequently exposed to oleic acid (OA, 50 μM) for 24 h. Representative images of LD staining (LipidGreen2). Nuclei were counterstained with DAPI (blue). Scale bar: 50 μm. (D) Quantification of LD in spinal cord astrocytes treated as indicated in (C). LD numbers were normalized by the number of nuclei analyzed (*n* = 3, mean ± SD). (E) Relative luminescence produced by firefly luciferase expressed under an NF‐κB‐driven promoter in cultures treated as indicated in (C). Relative firefly luciferase luminescence was corrected by the amount of *Renilla* luciferase activity controlled by a constitutive promoter and expressed as percentage of control cultures (NULL/BSA) (*n* = 4, mean ± SD). (F) Motor neuron survival determined 72 h after being plated on top of Ldha^flox/flox^ spinal astrocytes treated as indicated in (C) (*n* = 4, mean ± SD). ****p < 0.0001, **p < 0.01, *p < 0.05.

We next confirmed our findings using a genetic approach to inhibit LDHA in astrocytes, using astrocyte cultures from *Ldha*‐floxable mice. Astrocytes were transduced with a control adenovirus (null, empty open reading frame) or an adenovirus expressing Cre recombinase under the control of the CMV promoter, and 72 h later were subjected to FA overload. Genetic ablation of LDHA markedly decreased LD accumulation (Figure 6C,D) and attenuated NF‐κB‐dependent transcriptional activity relative to FA‐treated null controls (Figure 6E). Notably, LDHA deletion also mitigated FA‐induced neurotoxicity (Figure 6F).

**FIGURE 7:**
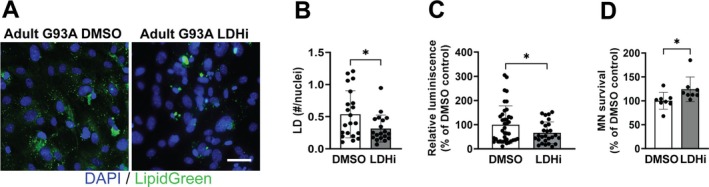
Lactate dehydrogenase inhibition ameliorates the neurotoxic phenotype of spinal cord astrocytes isolated from symptomatic hSOD1^G93A^ mice. (A) Representative LD staining (LipidGreen2) in spinal cord astrocyte cultures from symptomatic hSOD1^G93A^ (G93A) mice treated with GSK2837808A (2.5 μM, LDHi) or vehicle (DMSO) for 24 h. Nuclei were counterstained with DAPI (blue). Scale bar: 50 μm. (B) Quantification of LD normalized by the number of nuclei analyzed (*n* = 3, mean ± SD). (C) Relative luminescence produced by firefly luciferase expressed under an NF‐κB‐driven promoter in cultures treated as indicated above. Relative firefly luciferase luminescence was corrected by the amount of *Renilla* luciferase activity controlled by a constitutive promoter and expressed as percentage of cultures treated with vehicle (DMSO) (*n* = 3, mean ± SD). (D) Motor neuron survival in co‐cultures with spinal cord astrocytes from symptomatic hSOD1^G93A^ mice treated as in (A) (*n* = 4, mean ± SD). *p < 0.05.

**FIGURE 8:**
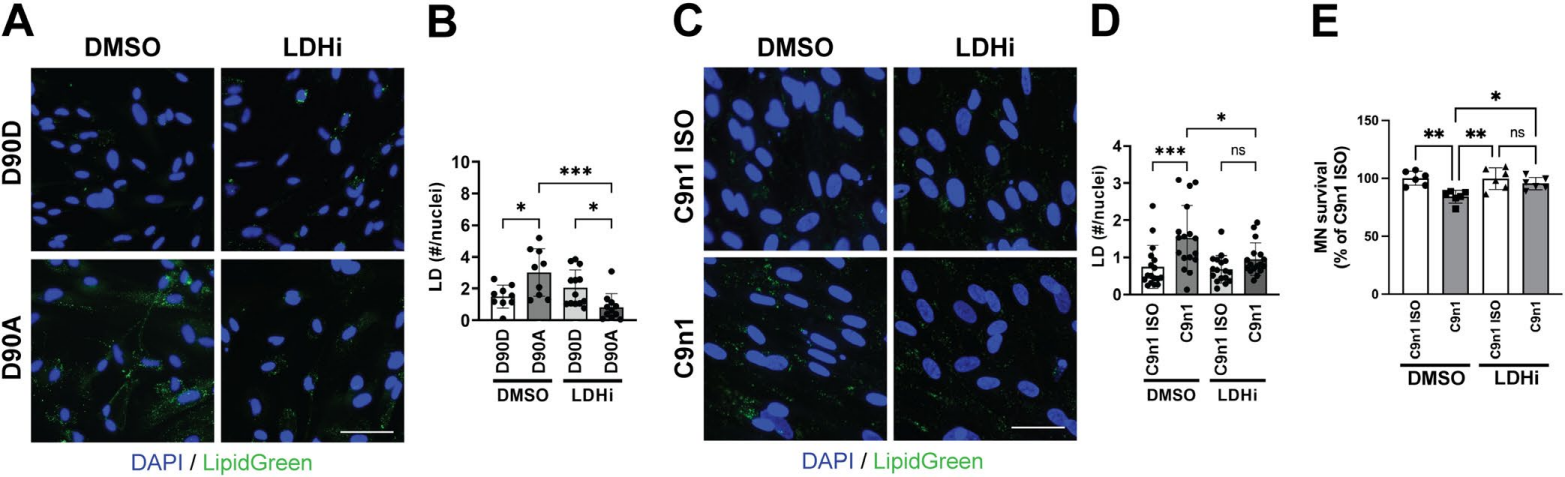
Lactate dehydrogenase inhibition decreases LD content in human inducible pluripotent stem cell‐derived astrocytes (iAs) from ALS patients. (A and C) LD (LipidGreen2) staining of iAs from SOD1^D90A^ (D90A)‐linked and C9orf72‐linked (C9n1) ALS patients and their corresponding isogenic corrected control, D90D and C9n1 ISO, respectively. Cultures were treated with GSK2837808A (2.5 μM, LDHi) or vehicle (DMSO) for 24 h. Nuclei were counterstained with DAPI (blue). Scale bar: 50 μm. (B and D) Quantification of the number of LD in the cultures shown in (A) and (C). LD number was normalized by the number of nuclei analyzed (*n* = 3, mean ± SD). (E) Motor neuron survival in co‐cultures with C9orf72‐linked (C9n1) ALS iAs or their corresponding isogenic corrected control, C9n1 ISO. iAs cultures were treated as in (A) 24 h before motor neuron plating (*n* = 3, mean ± SD). ***p < 0.001, **p < 0.01, *p < 0.05.

Astrocyte cultures obtained from the spinal cord of symptomatic hSOD1^G93A^ rats (Jimenez‐Riani et al. 2017) and mice (Figure 2A,B) display a greater number of LD when compared to nontransgenic astrocytes. Since it has been previously shown that these astrocytes are toxic to motor neurons in co‐culture and display a proinflammatory phenotype (Diaz‐Amarilla et al. 2011; Killoy et al. 2020), we tested the effect of inhibiting LDHA in these cells. LDHA inhibition in astrocyte cultures isolated from symptomatic ALS mice significantly decreased the accumulation of LD (Figure 7A,B) and decreased NF‐κB‐driven transcriptional activity (Figure 7C). In addition, this strategy increased the number of motor neurons that survive in co‐culture with these astrocytes (Figure 7C). In agreement with the observation made in astrocyte cultures from symptomatic ALS mice, human iPSC‐derived astrocytes from C9orf72‐linked and mutant SOD1‐linked ALS patients display a significant increase in the accumulation of LD when compared to their corresponding isogenic corrected control. In these cells, LDHA inhibition decreases the number of LD to the control levels (Figure 8A–D). Furthermore, this treatment increases the number of motor neurons that survive in co‐culture with C9orf72‐linked iPSC‐derived ALS astrocytes (Figure 8E).

## 4 Discussion

LD function as energy reservoirs while maintaining lipid homeostasis in the cell. In addition, LD buffer lipotoxicity, mainly by sequestering lipids containing polyunsaturated FAs, which are particularly vulnerable to peroxidation. Changes in LD dynamics could be a pathological hallmark, while at the same time contributing to the pathological process. Recent studies have proposed that one possible mechanism by which APOE4/4 is linked to increased risk for Alzheimer's disease may be due to its effects in LD dynamics in astrocytes and microglia that ultimately contribute to exacerbated lipid peroxidation and inflammation (Haney et al. 2024; Windham et al. 2024). Similarly, during macrophage activation, an increase in TAG synthesis and LD formation is essential for peak inflammatory macrophage activation (Castoldi et al. 2020). In addition, the accumulation of LD can also lead to the loss of function of hepatocytes in nonalcoholic fatty liver disease by acting as internal mechanical stressors (Loneker et al. 2023). Our data linking LD accumulation to the development of a neuroinflammatory and neurotoxic phenotype in astrocytes suggest that rather than solely function as a protective mechanism to neighboring neurons, the accumulation of LD is tightly coupled to a phenotypic transformation in astrocytes that affects astrocyte–neuron interaction.

In astrocytes, LD accumulation has been associated with various stress stimuli (Smolic et al. 2021). In cultured astrocytes, expression of ALS‐linked mutations leads to LD accumulation (Jimenez‐Riani et al. 2017; Miquel et al. 2024; Velebit et al. 2020). In addition, we showed that LD accumulation is observed in vivo in two different mutant SOD1‐linked ALS mouse models. Of note, while this manuscript was under review, Marcadet et al. (2025) observed the accumulation of LD in spinal cord astrocytes from a FUS‐linked ALS model and in postmortem tissue samples from individuals carrying ALS‐linked FUS mutations. Further stressing the relevance of our observations to the human pathology, we showed that LD accumulation also occurs in two different lines of iPSC‐derived astrocytes from ALS patients harboring a SOD1 mutation or a C9orf72 expansion. In these lines, the increase in LD number is associated with the expression of the ALS‐linked mutations, since the reversion to the wild‐type allele in the isogenic corrected controls decreases the number of LD.

Our observations in the spinal cord of ALS mice indicate that the presence of LD is not restricted to astrocytes, but is also present in other cell types, likely including neurons and microglia. This finding suggests that altered LD homeostasis in these cells may also play a crucial role during disease progression and warrants further investigation. Taken together, our data suggest that the accumulation of LD may be a common pathological feature of ALS. Moreover, since the formation of LD is a well‐known indicator of a change in cell metabolism (Olzmann and Carvalho 2019), our results provide additional evidence to the notion that altered energy balance may contribute to neuronal death in ALS (Ludolph et al. 2023).

In activated immune cells, the formation of LD is well known to contribute to the production of inflammatory lipid mediators (den Brok et al. 2018; Jarc and Petan 2020). These mediators can elicit inflammatory responses in immune cells through autocrine or paracrine mechanisms. In astrocytes, FA loading and LD formation may lead to a similar mechanism, where increased synthesis of inflammatory mediators leads to increased NF‐κB‐driven transcriptional activity. The exact contribution of metabolic changes to the phenotype of reactive astrocytes is not yet fully understood. However, it seems clear that astrocytes undergo metabolic reprogramming to support a proinflammatory response (Robb et al. 2020; Xiong et al. 2022). Thus, the proinflammatory response that accompanies LD accumulation may be supported by an increase in aerobic glycolysis that can be thwarted by LDH inhibition. Alternatively, LDH inhibition in astrocytes may lead to the breakdown of LD and the transport of FAs to the mitochondria to sustain oxidative phosphorylation in lieu of aerobic glycolysis. This will lead to a corresponding weakening of the stressor mechanism (e.g., accumulation of LD) while it may not have major deleterious consequences for astrocytes, since these cells have efficient detoxification and antioxidant defenses to counteract mitochondrial reactive oxygen species production during β‐oxidation (Vargas and Johnson 2009). In fact, astrocytes play a pivotal role in FA metabolism and loss of FA degradation capacity by astrocytes triggers neuroinflammation and neurodegeneration (Mi et al. 2023).

Even in conditions of normoxia, astrocytes are believed to have a higher glycolytic rate than neurons (Dringen and Hamprecht 1993; Supplie et al. 2017; Swanson and Benington 1996), with the resulting lactate serving as an energy source for coupled neurons via the astrocyte–neuron lactate shuttle (ANLS) (Herrero‐Mendez et al. 2009; Sotelo‐Hitschfeld et al. 2015). Astrocyte‐derived lactate may be critical in some scenarios. However, in our culture conditions, inhibiting LDH in astrocytes had no deleterious effect for co‐cultured motor neurons in control conditions. While the ANLS is a foundational concept, the strict dependence of neurons on astrocyte‐derived lactate and the validity of the stoichiometry of the process remain contested (Diaz‐Garcia et al. 2017; Dienel 2017; Patel et al. 2014). In our co‐cultures, inhibiting astrocytic glycolysis could theoretically compromise neuronal support. However, our results demonstrate that motor neuron survival remains unchanged whether astrocytes are treated with a chemical LDH inhibitor or undergo genetic *Ldha* ablation (Figures 5E,F, 6F, and 8E). These data suggest that, at least in optimized culture conditions, motor neurons do not critically rely on astrocyte‐derived lactate. We propose that the protective effects of LDH inhibition—specifically the promotion of LD clearance and the attenuation of proinflammatory signaling—outweigh the potential loss of lactate fueling. We note, however, that our optimized culture conditions may mask metabolic dependencies that could emerge under nutrient‐limited conditions in vivo. In summary, our data show that inhibition of LDHA counteracts LD accumulation and the associated neurotoxic phenotypic transformation of astrocytes.

## Author Contributions

D.E., M.B., S.R., and D.O. performed experiments. D.E., M.P., and M.R.V. analyzed data. D.E., M.P., and M.R.V. wrote the paper. All authors reviewed and approved the content of the manuscript.

## Funding

This work was supported by the National Institute of Neurological Disorders and Stroke (R01NS122973, R01NS089640).

## Conflicts of Interest

The authors declare no conflicts of interest.

## Supporting information


**Figure S1:** Lipid droplet content in the ventral horn of the spinal cord from wild type hSOD1^WT^. Perilipin 2 (PLIN2, red), LipidGreen2 (green), and GFAP (magenta) staining in lumbar spinal cord sections of age‐matched nontransgenic (NonTG) and wild‐type hSOD1 (SOD1WT) mice. Nuclei were counterstained with DAPI (blue). Scale bar: 50 μm Quantification of the number of LD present in 50,000 μm^2^ area of the ventral horn in the spinal cord from SOD1WT mice is shown in Figure 1E.

## Data Availability

The data that support the findings of this study are available from the corresponding author upon reasonable request.
