# Supplementary material for: Lactate Dehydrogenase Inhibition Reverts the Fatty Acid‐Induced Neurotoxic Phenotype of Astrocytes
Source: Glia. 2026 Jan 6;74(3):e70136. doi: 10.1002/glia.70136 (PMC12772936; doi:10.1002/glia.70136)
Supplement: Supplementary file 1 — Figure S1: Lipid droplet content in the ventral horn of the spinal cord from wild type hSOD1WT. Perilipin 2 (PLIN2, red), LipidGreen2 (green), and GFAP (magenta) staining in lumbar spinal cord sections of age‐matched nontransgenic (NonTG) and wild‐type hSOD1 (SOD1WT) mice. Nuclei were counterstained with DAPI (blue). Scale bar: 50 μm Quantification of the number of LD present in 50,000 μm2 area of the ventral horn in the spinal cord from SOD1WT mice is shown in Figure 1E. [file GLIA-74-0-s001.docx]

**
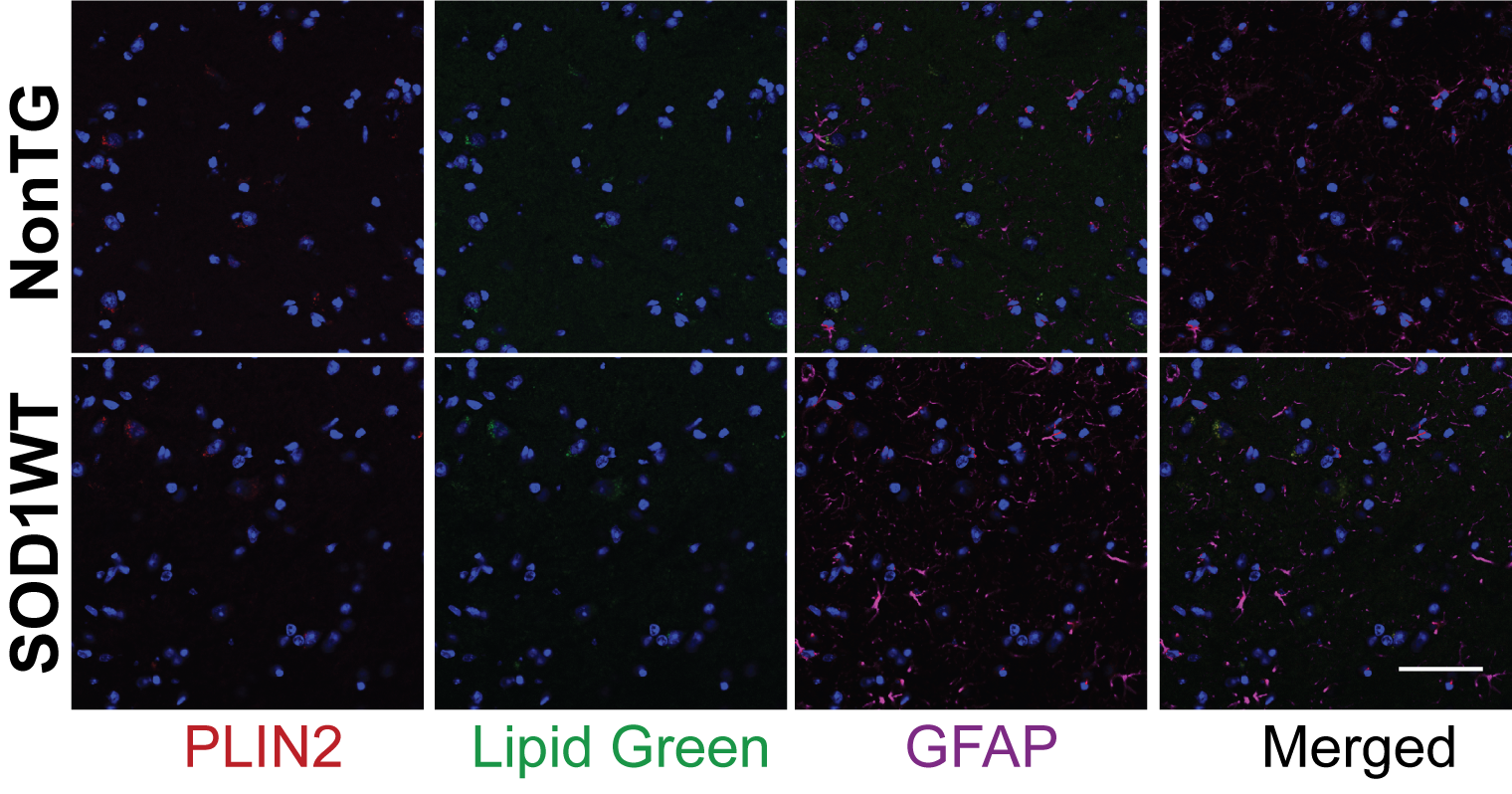
**

**Supplementary Figure 1. Lipid droplet content in the ventral horn of the spinal cord from wild type hSOD1^WT.^** Perilipin 2 (PLIN2, red), LipidGreen2 (green), and GFAP (magenta) staining in lumbar spinal cord sections of age-matched non-transgenic (NonTG) and wild type hSOD1 (SOD1WT) mice. Nuclei were counterstained with DAPI (blue). Scale bar: 50 µm Quantification of the number of LD present in 50000 µm^2^ area of the ventral horn in the spinal cord from SOD1WT mice is shown in Figure 1E.
